# Occupational Asthma and Contact Urticaria in a Wildlife Worker With Type I Hypersensitivity to Deer Dander

**DOI:** 10.1111/cod.70136

**Published:** 2026-03-20

**Authors:** Eglė Janušonytė, Thomas Harr, Peter Jandus, Haïg Nigolian

**Affiliations:** ^1^ Division of Immunology and Allergology Geneva University Hospitals Geneva Switzerland; ^2^ Department of Dermatology and Venereology Geneva University Hospitals Geneva Switzerland

**Keywords:** deer allergy, occupation allergy, occupational contact urticaria

1

Occupational exposure to animal dander is a well‐recognised cause of IgE‐mediated respiratory and cutaneous allergies, particularly among farmers, veterinarians and laboratory workers. However, allergy to cervid dander remains rarely documented, with only a handful of cases reported in the literature. We present the case of a wildlife professional who developed occupational contact urticaria, rhinoconjunctivitis, and asthma following exposure to deer.

## 2 Case Report

A 36‐year‐old male patient presented with recurrent localised wheals occurring after contact with cervid dander. His professional activity involved wildlife management, including deer culling, carcass handling and GPS collaring. On several occasions, localised wheals appeared within minutes of direct exposure to blood droplets during evisceration.

The patient also reported itchy, watery eyes and nasal congestion with runny nose, as well as worsening of pre‐existing asthma during cervid‐related activities, but did not experience other systemic reactions or angioedema. He had never experienced any reactions after eating cooked meat and regularly consumed venison, mainly red deer ( *Cervus elaphus* ) and roe deer ( *Capreolus capreolus* ), in various processed forms (minced steaks, sausages, dry‐cured sausages) as well as in minimally processed preparations such as stews and seared fillet (tataki‐style). The meat was sometimes eaten very rare, and he also recalled previous consumption of red deer ( *Cervus elaphus* ) tartare. More recently, he had eaten a burger prepared with a rare to medium‐rare ground elk ( *Cervus canadensis* ) patty.

No symptoms occurred upon regular exposure to other wild animals such as wild boars, foxes, badgers, hedgehogs and squirrels, nor upon contact to his domestic pets (three dogs and a python). As a child, he had received allergen immunotherapy for horse, cats, and rabbit type I hypersensitivity.

Skin prick tests were performed using a standard panel of inhalant and epithelial allergens routinely used in our allergy unit, which the following returned positive results: *Dermatophagoides pteronyssinus*, *Dermatophagoides farinae* (Lofarma SpA, Milan, Italy), *Alternaria alternata* , *Cladosporium herbarum* (Stallergenes‐Greer International, Baar, Switzerland), *Betula verrucosa* , *Phleum pratense* , *Fraxinus excelsior* , dog, and cat (Allergopharma GmbH & Co, Reinbek, Germany). Histamine at 1 mg/mL was used as a positive control and saline solution as a negative control (Allergopharma GmbH & Co, Reinbek, Germany). Wheal size was measured after 15 min, and a test was considered positive when the allergen‐induced wheal was at least as large as the histamine‐induced wheal.

In accordance with EAACI recommendations for prick‐to‐prick testing with native materials [1], animal dander was prepared from raw material consisting of hair and dander from red deer ( *Cervus elaphus* ; sample collected from a wild animal), roe deer ( *Capreolus capreolus* ; sample collected from a wild animal), and European fallow deer ( *Dama dama* ; sample collected from a captive animal). The material was extracted in 0.9% saline and ground in a sterile mortar; lancets were then coated with the resulting suspension and applied directly to the skin.

**FIGURE 1:**
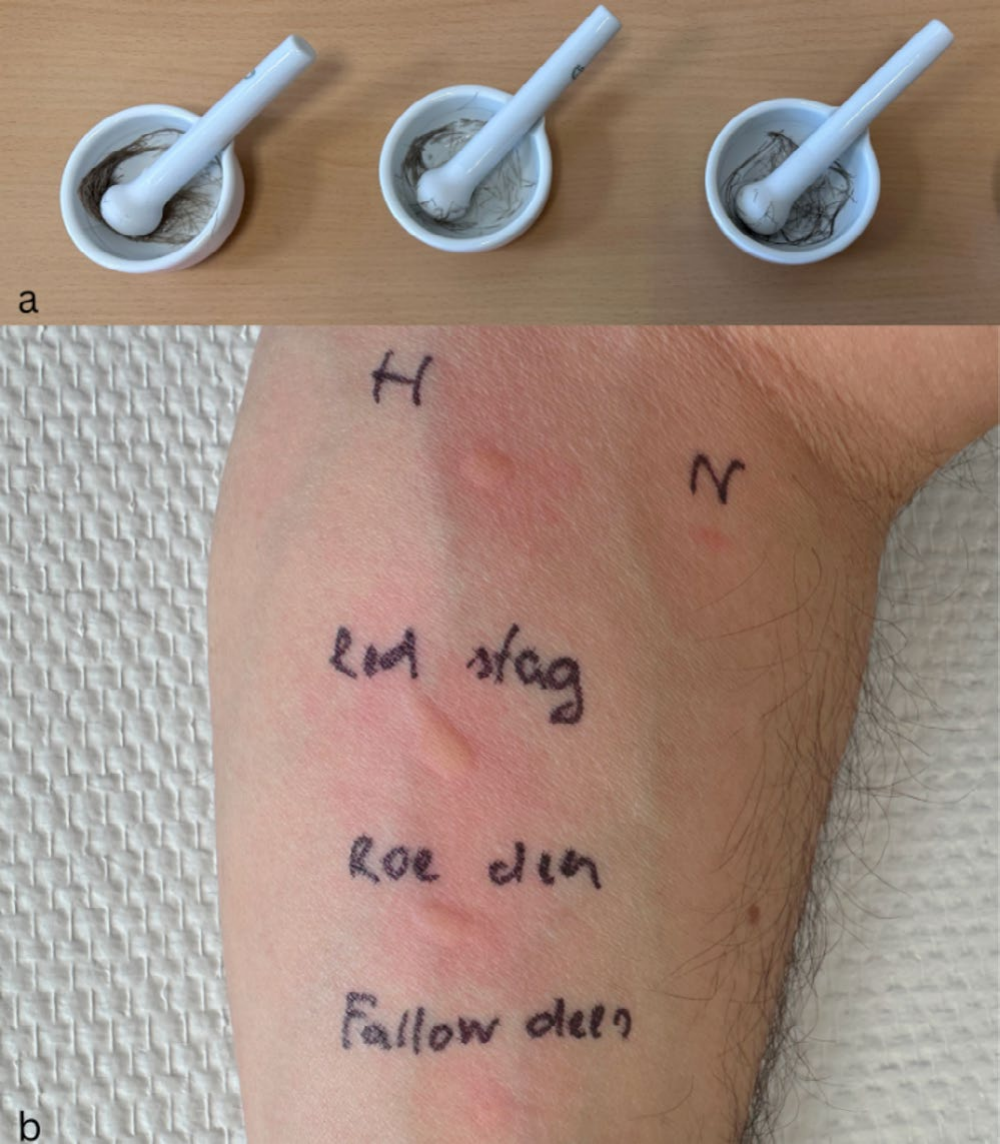
Prick‐to‐prick testing with cervid dander in volar forearm. From proximal to distal the tested materials are red deer ( *Cervus elaphus* ), roe deer ( *Capreolus capreolus* ), and fallow deer ( *Dama dama* ): Wheal reactions (≥ 10, ≥ 7 and ≥ 5 mm) were measured (b) including histamine (H) and saline NaCl (N) controls.

This testing induced strongly positive wheal reactions (≥ 10, ≥ 7 and ≥ 5 mm above the negative control for red deer, roe deer and fallow deer, respectively) (Figure 1). The histamine control produced a 6 mm wheal, whereas the saline control was considered negative, as no palpable papule was detected and only erythema was observed.

The control group consisted of eight non‐atopic hospital staff members, all of whom tested negative for the same allergens, confirming that the findings were not attributable to irritant responses.

In vitro multiplex testing (ALEX2, Macro Array Diagnostics, Vienna, Austria) confirmed broad sensitization to aeroallergens, with specific reactivity to animal lipocalins from cats (Fel d 4), dogs (Can f 1), bovine (Bos d 2), and horses (Equ c 1), as well as dog albumin (Can f 3) and cat uteroglobin (Fel d 1).

Briefly, ALEX2 is a multiplex assay that evaluates sIgE responses to 295 individual allergens immobilised on a nitrocellulose membrane [2]. Patient serum is incubated with the membrane, and after enzymatic processing, signal intensity is converted into quantitative results expressed in kUA/mL [2].

Based on clinical history and diagnostic findings, a diagnosis of cervid dander allergy with contact urticaria, rhinoconjunctivitis, and occupational asthma was established. The patient reported reproducible deterioration of respiratory function, including chest tightness and wheezing, specifically during and shortly after cervid‐related activities, with complete symptom resolution following exposure cessation. Although baseline spirometry performed outside periods of deer exposure did not show airflow obstruction, the clear temporal association between cervid exposure and respiratory onset, in conjunction with a markedly elevated fractional exhaled nitric oxide (FeNO 58 ppb; normal < 25 ppb) indicative of eosinophilic airway inflammation, supported the diagnosis of occupational asthma.

Management focused on reducing cervid exposure, including both live animals and carcasses, and the use of personal protective equipment, such as gloves and masks during professional activities. In addition, occupational adjustments were recommended to further reduce future risk, including strict avoidance of cervid exposures. The patient was prescribed oral antihistamines and inhaled budesonide/formoterol and was also equipped with an emergency kit containing an adrenaline (epinephrine) auto‐injector.

## 3 Discussion

Occupational deer allergy is a rare condition and scarcely documented in the literature. Nahm et al. described a farmer who developed occupational asthma and rhinitis after raising three red deer, confirmed by positive skin‐prick testing with deer dander extract [3]. Spiewak et al. reported contact urticaria in a professional hunter after exposure to roe deer fur, confirmed by a rub test [4]. Similarly, Carballada et al. described two workers at animal rehabilitation centres who were sensitised to roe deer [5]. One patient had a history of rhinoconjunctivitis and the other a history of rhinoconjunctivitis and probable asthma. Both underwent skin testing with a standard panel of inhaled and epithelial allergens as well as with roe deer hair extract, revealing positive reactions for both roe deer hair and dander extracts and positive conjunctival provocation tests with roe deer hair extract [5]. In addition, an occupational exposure to deer‐associated ectoparasites has also been documented in a 42‐year‐old research assistant working in a geological institute who developed IgE‐mediated rhinoconjunctivitis to deer ked, a type of louse fly [6].

Beyond occupational settings, residential exposure has also been reported. Amrol et al. described a 4‐year‐old boy who experienced hives, swelling, and shortness of breath within minutes after exposure to a pet deer kept in a friend's house [7]. IgE‐mediated sensitization was confirmed by positive prick testing to deer [7].

Gillespie et al. studied 15 highly atopic individuals with historic or current sensitization to deer or elk. All 15 had concurrent reactivity to at least one domestic animal dander (dog, cat, or horse) [8]. Elevated IgE to deer hair/dander was detected in 12 patients, while 6 demonstrated elevated IgE to elk hair/dander [8]. RAST inhibition experiments further demonstrated shared allergens across hair/dander, urine, and serum fractions, with one patient showing cross‐reactivity between deer and cat dander, indicating possible IgE antibody cross‐reactivity between deer elk and other mammalian allergens in sensitised individuals [8].

A limitation of this case is that skin prick testing or specific IgE measurement to cervid meat or blood was not performed. Such testing would have further characterised the patient's sensitization profile and provided insight into his tolerance of ingested venison despite cutaneous reactivity to dander.

Despite this limitation, our case highlights an uncommon but clinically relevant presentation of cervid allergy, combining urticaria, rhinoconjunctivitis, and asthma. Prick‐to‐prick testing remains an underused diagnostic tool but can be particularly valuable in occupational allergies, where standardised extracts are often unavailable. Although such conditions are rare, systematic use of this method should be encouraged to improve recognition and diagnosis. Greater awareness of this occupational entity is essential, as underdiagnosis may result in ongoing exposure and disease progression in wildlife professionals.

## Author Contributions

**Eglė Janušonytė:** conceptualization, writing – original draft, investigation, writing – review and editing. **Thomas Harr:** writing – review and editing. **Peter Jandus:** writing – review and editing. **Haïg Nigolian:** conceptualization, writing – original draft, investigation, writing – review and editing.

## Funding

The authors have nothing to report.

## Consent

Written informed consent was obtained from the patient for publication of this case and accompanying image.

## Conflicts of Interest

The authors declare no conflicts of interest.

## Data Availability

The data that support the findings of this study are available from the corresponding author upon reasonable request.
